# Exploring marine glycans: structure, function, and the frontier of chemical synthesis

**DOI:** 10.1039/d5cb00090d

**Published:** 2025-06-04

**Authors:** Sandhya Mardhekar, Phuong Luong, Peter H. Seeberger

**Affiliations:** a Department of Biomolecular System, Max Planck Institute for Colloids and Interfaces Am Muhlenberg 1 14476 Potsdam Germany peter.seeberger@mpikg.mpg.de; b Institute for Chemistry and Biochemistry, Freie Universität Berlin Arnimallee 22 14195 Berlin Germany

## Abstract

Marine glycans are structurally diverse biomolecules that play pivotal roles in oceanic carbon cycling by regulating microbial metabolism, accelerating organic matter turnover, and contribute to carbon sequestration. Glycans originating from marine organisms exhibit a wide range of bioactivities and applications in medicine, biotechnology, cosmetics, food and agriculture. The structural complexity of glycans poses significant challenges in understanding their functions, as traditional purification and characterization methods are often hindered by their inherent heterogeneity. To overcome these challenges, enzymatic extraction using glycoside hydrolases and carbohydrate-active enzymes (CAZymes) enables the selective recovery of native glycans, while automated glycan assembly (AGA) provides a robust approach for the rapid and reproducible synthesis of structurally defined glycans. Subjecting synthetic glycans to enzymatic degradation enables researchers to explore the inverse relationship between glycan complexity and microbial degradation, suggesting that algae can generate complex glycans at a rate exceeding bacterial decomposition, thereby reinforcing carbon storage. Here, we present a comprehensive overview of marine glycan sources and their structural diversity. We highlight the importance of employing two complementary methods, enzymatic extraction as a critical tool for glycan identification and AGA as an advanced synthetic platform, to build a refined framework for elucidating the ecological role and industrial potential of marine glycans.

## Abstract 2

This review presents a high-level overview of marine glycans, highlighting their structural complexity, functional diversity, and emerging chemical methods for their analysis and synthesis.

## Graphical abstract

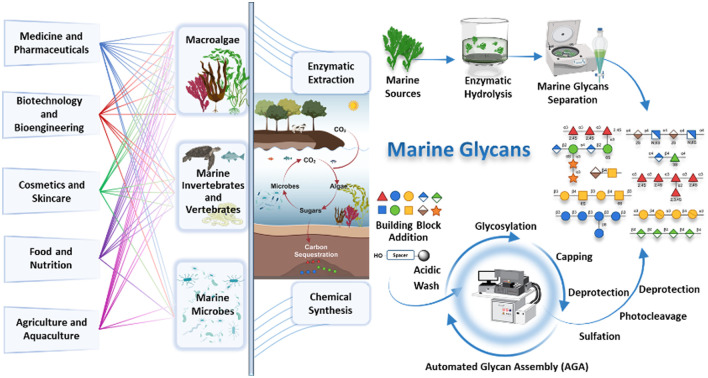

## 1. Introduction

The ocean fixes approximately 50 gigatons of carbon each year, making it the Earth's most important carbon reservoir.^1^ This immense carbon storage is regulated by the marine carbon cycle, a crucial process that maintains the global carbon balance. Central to this cycle are glycans, or complex polysaccharides that fuel carbon fixation and transfer. Microscopic planktonic algae, such as diatoms, capture carbon dioxide from the atmosphere at the ocean's surface and convert it into organic matter in the form of glycans.^2,3^ These glycans provide structural integrity to the algal cell walls^4,5^ and are subsequently circulated through the marine food web as organisms consume algae and other plankton. When marine organisms die, their remains form sinking particles that release glycans into the environment. While these glycans are typically degraded by deep-sea microbes, many are resistant to decomposition and remain sequestered in the ocean floor for up to centuries.^6,7^

Beyond their ecological importance, marine glycans have diverse applications across various industries (Table 1). The therapeutic potential of marine glycans has been explored for anti-cancer^8^ and anti-microbial^9^ activity as well as for drug delivery enhancement.^10^ In biotechnology and bioengineering, marine glycans are utilized for bioprinting,^11^ tissue engineering,^12^ and other innovative technologies. In the cosmetics and food industries, these glycans serve as active ingredients in cosmetics^13^ and key components for food packaging^14^ and preservation.^15^ In agriculture and aquaculture, marine glycans have been employed to improve fertilization,^16^ plant growth,^17^ and animal health.^18^ The range of application for marine glycans continue to expand in numerous fields.

**Table 1:** Industrial applications of marine glycans

Glycan	Medicine and pharmaceutical	Biotechnology and bioengineering	Cosmetics and skincare	Food and nutrition	Agriculture and aquaculture
Alginate	Anti-microbial,^9,89–91^ anti-cancer,^92,93^ anti-fibrosis,^94^ drug delivery^10^	Bioprinting,^11,95,96^ tissue engineering,^12,97,98^ fluorescent materials,^99^ hydrogels,^100^ bioplastic,^99^ air-filtration^101^	Scrubbing additive,^102^ exfoliating agent,^103^ active ingredient-carriers^13,104,105^	Edible films,^106,107^ food packaging^14,108,109^	Irrigation,^110^ fertilization,^16^ desalination,^111^ herbicide^112^ and pest control^113,114^

Fucoidan	Anti-cancer,^8,115,116^ anti-diabetes,^117–119^ neuroprotective,^120^ vaccination,^121^ gut microbiota regulation^122^	Bioprinting,^123^ bone tissue engineering,^124^ endothelialization,^125–127^ biomaterials^128,129^	Whitening,^130^ antioxidant,^131–133^ skin protection,^134^ cosmetics formulation^135^	Food addictive delivery,^136^ digestibility,^137^ starch quality^138^	Animal health,^18,139^ pesticide toxicity reducing^140^

Laminarin	Anti-bacterial,^141^ anti-cancer,^142,143^ immunomodulatory,^144,145^ gut microbiota regulation^146,147^	Membrane anti-fouling,^148^ bioplastic^149^	Anti-wrinkle,^80^ skin protection^150,151^	Shelf-life extension,^15^ digestibility,^152^ gelatinization^153^	Fish immunopotentiator^154,155^

Carrageenan	Anti-viral,^156^ anti-cancer,^157^ anti-inflammatory^158^	Bioprinting,^72,159^ bone tissue engineering,^160–162^ aerogel,^163^ hydrogels,^164^ biodegradable devices^165^	Skin hydration,^81^ anti-photoaging^166^	Cold storage,^167^ meat quality,^168,169^ anti-glycation^170^	Plant growth,^17,171^ fertilization,^172^ pest control,^173^ dye removal^174^

Agar	Drug delivery,^175^ nanomedicine^176^	Bioprinting,^177^ bio-cleaning,^178^ hydro-films^179^	Wound healing,^180^ anti-aging facemasks^181^	Emulsifier,^182^ food jellies,^87^ packaging^183,184^	Harvesting,^185^ dye removal^186^

Mannan	Anti-viral,^66^ anti-cancer^187^				

Ulvan	Anti-viral,^188^ anti-inflammatory,^189^ anti-diabetes,^190^ immunomodulatory,^67^ drug delivery^191,192^	Skin tissue engineering,^193^ endothialization,^194^ nanofibers,^195,196^ hydrogels^197,198^	Antioxidant and whitening^82^	Edible films,^199^ healthy-aging nutraceuticals^200^	Plant health,^201,202^ fish immunopotentiator^203^

Chitin	Anti-microbial,^204^ anti-cancer,^68,205^ anti-inflammatory^206^	Bioprinting,^74^ hydrogels,^207,208^ nanomaterials^209,210^	Skin regeneration^211,212^	Emulsifier,^213^ packaging,^214,215^ quality control^216^	Pest control,^217^ metal^218^ and microplastic^219^ removal

Chitosan	Anti-viral,^69,220^ anti-bacterial,^221,222^ anti-cancer,^223^ anti-coagulant^224^	Green nanotechnology,^75^ biosensor,^225,226^ hydrogels^227^	Anti-aging,^83,228^ color cosmetics,^229^ skin penetration,^230^ acne removal^231^	Packaging,^232,233^ quality control^234^	Soil strength,^235^ fertilization,^236^ pest control,^237,238^ wastewater filtration,^239,240^ plant health^241,242^

GAGs	Anti-inflammatory,^243,244^ cartilage repair,^245,246^ bone regeneration,^247^ neuroprotective^248^	Brain tissue repair,^249^ 3D biomimetics,^250,251^ tissue engineering^252^	Anti-aging and antioxidant,^84^ cosmetics formulation^253^	Functional food ingredients^254–256^	

Glucan	Anti-cancer,^257,258^ immunomodulatory,^58^ intestinal health^57^				Fish immunopotentiator^259,260^

EPS	Anti-bacterial,^71^ anti-cancer,^261,262^ anti-coagulant,^263^ anti-inflammatory^264^	Biodiesel,^265^ biosurfactants,^266^ bioremediation^77^	Anti-allergy,^85^ active ingredient formulation^86^	Emulsifier,^267,268^ thermostable gelling agent^269^	Aquatic infection control,^270^ metal removal^271^

The primary challenge associated with marine glycans lies in their structural complexity. While their functional roles are well-established, the detailed molecular mechanisms by which their structures dictate these functions remain poorly understood. This knowledge gap arises largely because most studies have been conducted using isolated glycans or heterogeneous mixtures, which provide bioactivity information but lack the precision needed to elucidate structure–function relationships. Although a limited number of studies have investigated these glycans through defined structures, such studies are often resource-intensive, requiring expert chemists and complex synthetic methodologies. As a result, advancing research techniques is imperative to deepening our understanding of marine glycans.

One approach to studying marine glycans is enzymatic extraction using CAZymes, enzymes that break down carbohydrates and provide insights into the building blocks of complex glycans.^19–22^ However, a limitation of this method is the lack of a full set of enzymes capable of degrading all glycans, highlighting their inherent complexity. This complexity likely explains their role in carbon sequestration, as some glycans resist microbial breakdown, contributing to long-term carbon storage in the ocean.^23,24^

To address this challenge, AGA has emerged as a powerful tool. AGA allows for the efficient, reproducible, and controlled synthesis of marine glycans, bypassing the need for expert chemists.^25,26^ Using AGA, researchers can create pure, defined glycans, enabling detailed studies such as NMR spectroscopy to identify specific epitopes and examine the enzymatic processes of carbohydrate degradation. This strategy helps uncover glycans that are resistant to microbial breakdown and their role in carbon sequestration. In particular, algae produce complex glycans with modifications, such as sulfation, that protect them from degradation and enhance their contribution to carbon storage in the ocean.

**Fig. 1:**
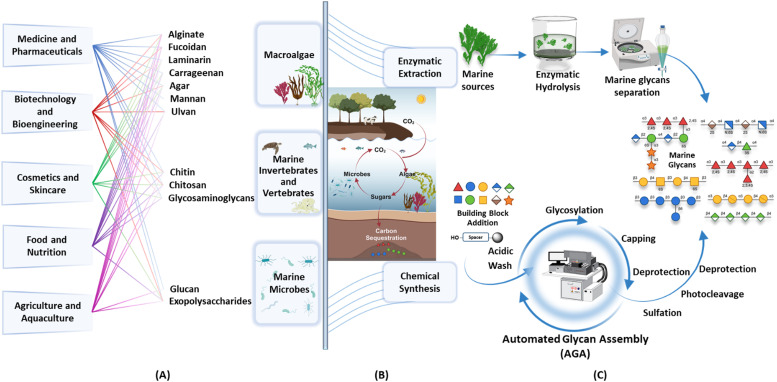
Overview over marine glycans and methods to establish their structure–activity relationships. (A) Marine glycans, sources, and applications. (B) Glycans as central metabolic fuels in the marine carbon cycle. (C) Enzymatic extraction and automated glycan assembly as complementary methods to access marine glycans.

Here, we review the structures and sources of marine glycans, while exploring the diverse bioactivities of glycans and their applications. We highlight two complementary approaches to gaining a deeper understanding of marine glycans, focusing on their role in the carbon cycle and their structure–function relationships. We emphasize the synergy between harnessing the industrial potential of marine glycans and advancing our molecular understanding of these complex molecules (Fig. 1).

## 2. Sources and structures of marine glycans

**Fig. 2:**
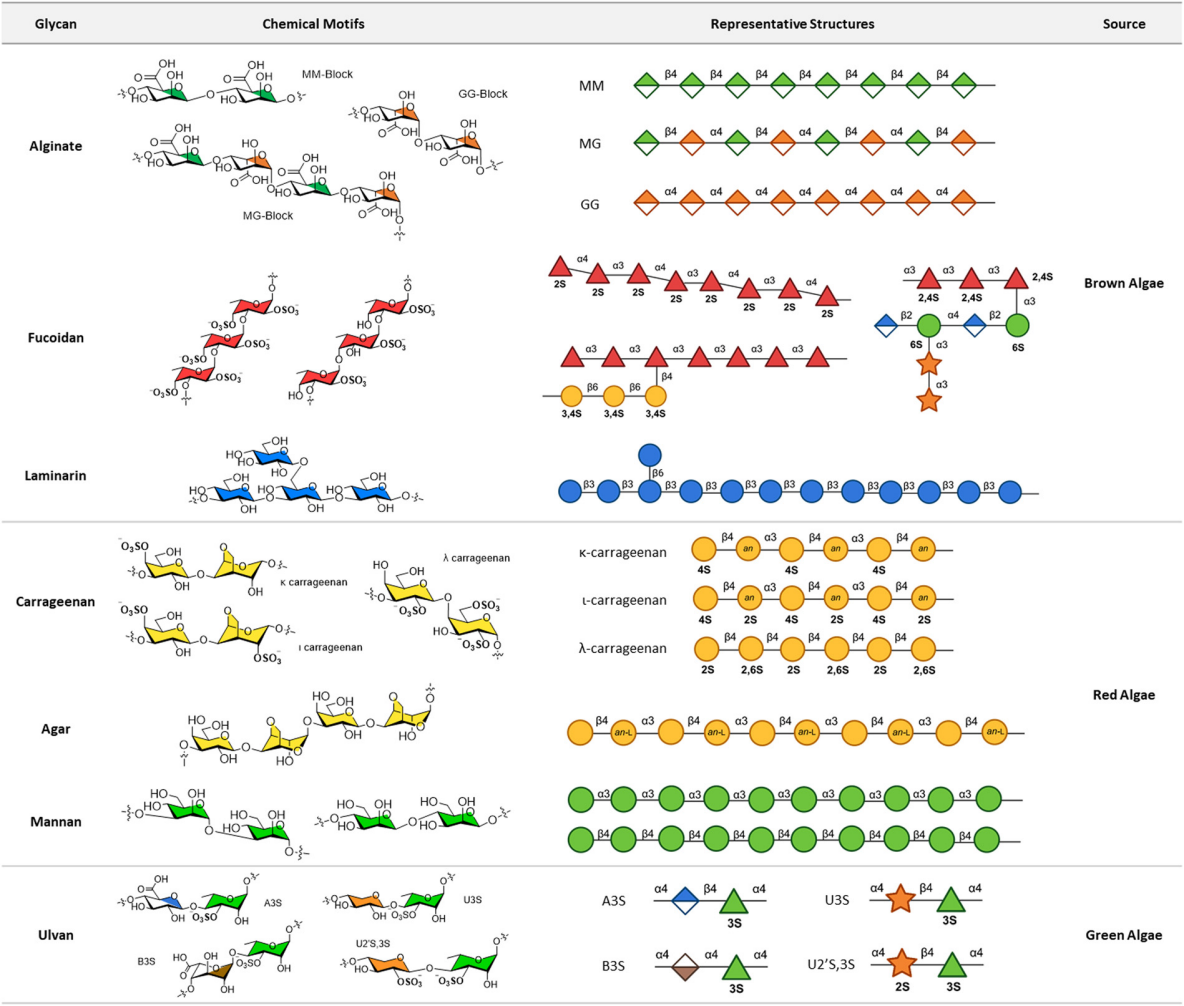
Structures of marine glycans derived from macroalgae. Chemical motifs highlight core structural features specific to each glycan, while the representative structures illustrate the diversity of these glycans in nature. All glycan symbols follow the symbol nomenclature for glycans (SNFG) guidelines.

**Fig. 3:**
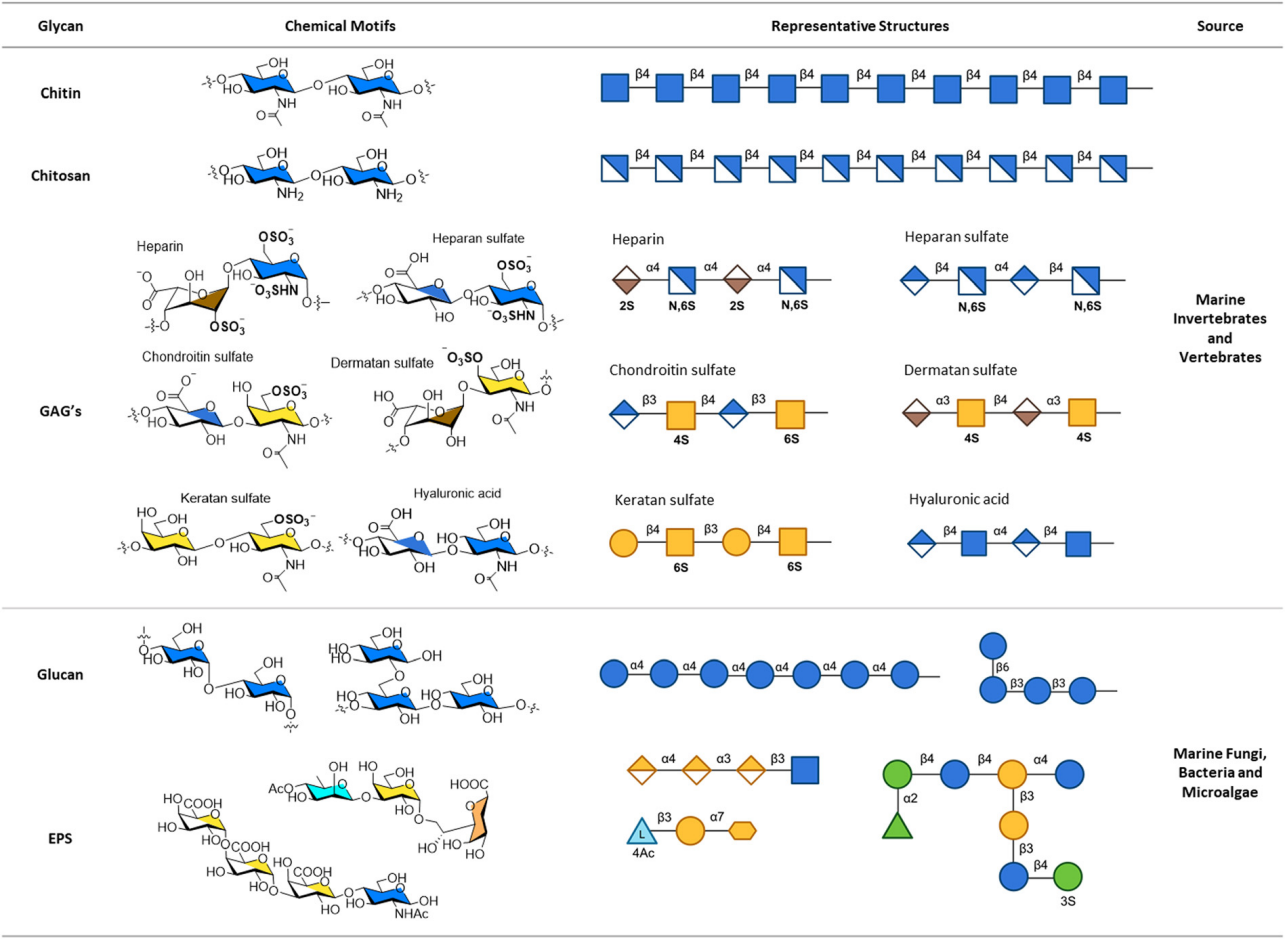
Structures of marine glycans derived from marine invertebrates, vertebrates, and microorganisms. Chemical motifs highlight core structural features specific to each glycan, while the representative structures illustrate the diversity of these glycans in nature. All glycan symbols follow the symbol nomenclature for glycans (SNFG) guidelines.

Marine organisms synthesize glycans that are vital for biological processes such as metabolism, cell signalling, immune modulation, and structural integrity.^5,27^ The structure and composition of these glycans vary significantly across classes of organisms and between species. In this section we categorize glycans from three major groups of marine organisms: macroalgae, invertebrates and vertebrates, and microbes (fungi, bacteria, and microalgae, including cyanobacteria and diatoms). The unique glycan structures within these groups will be explored in more detail to highlight their functional significance in the marine environment (see Fig. 2 and 3).

### 2.1. Glycans in marine macroalgae

Marine macroalgae, including brown, red, and green seaweeds, are vital components of the marine ecosystem. Each algal class produces unique glycans in their cell walls that contribute to structural integrity and facilitate cellular signalling.^4,5^ As crucial players in the marine carbon cycle, macroalgae generate diverse and complex glycans that resist microbial degradation, and enhance carbon sequestration in the ocean.^6^

Brown algae contain three main classes of glycans: alginate, fucoidan, and laminarin. The structure and composition of these glycans are highly species-specific and reflect the glycan diversity within brown algae.

#### 2.1.1 Alginate

Alginates are a major component of the extracellular matrix in brown algae. These linear polymers consist of three main units: β-(1 → 4)-d-mannuronic acid (M block), α-(1 → 4)-l-guluronic acid (G block), and the alternating β-(1 → 4)-d-mannuronic acid-α-(1 → 4)-l-guluronic acid (MG block) units. Alginates are typically found in calcium salt form, particularly in the G-rich regions^28^ and extracted from various brown seaweeds, including *Laminaria hyperborea*, *Laminaria digitata*,^29^ and *Macrocystis pyrifera.*^30^ The proportion of M and G blocks can vary depending on the species, with the G block content ranging from 10% to 70%.^31^

#### 2.1.2 Fucoidan

Fucose-containing sulfated polysaccharides, termed fucoidan, constitute a major component of the brown algal cell wall. These polysaccharides can be classified into two main types: homogeneous fucoidans, also known as fucans, which are primarily composed of highly sulfated l-fucose residues; and heterogeneous fucoidans that have more complex backbones incorporating monosaccharides other than fucose, such as d-galactose, d-xylose, d-mannose, and d-glucuronic acid.^28^ Fucans typically have two major backbone structures: one consisting of pure α-(1 → 3)-l-fucose and the other of alternating α-(1 → 3)-l-fucose and α-(1 → 4)-l-fucose linkages. The prevalence of these backbones varies by species, with Fucales (such as *Fucus* and *Sargassum*) being rich in the α-(1 → 3)-l-fucose backbone,^32^ while Laminariales are more enriched in the alternating backbone.^33^ Fucans are heavily sulfated on the l-fucose residues, with *O*-sulfate esters typically occupying the 2S, 3S, and 4S positions. In contrast, heterogeneous fucoidans display greater diversity in their backbones and degree of sulfation, with no single common structure or trait defining them. The full characterization of fucoidans remains incomplete, and further research is necessary to better understand their structural diversity.^28^

#### 2.1.3 Laminarin

Laminarin is a major component found in the vacuoles of brown algae, serving as a food reserve.^34^ Laminarin is typically characterized as a linear polymer of β-(1 → 3)-d-glucose units, with fractional branches of β-(1 → 6)-d-glucose residues. The ratio of (1 → 3) and (1 → 6) linkages varies between species. For instance, when extracted from *Dictyota dichotoma* and *Sargassum fusiforme*, the ratio is 3 : 1 for (1 → 3) and (1 → 6) linkages, while in *Sargassum duplicatum*, the ratio is 6 : 1.^35^ Laminarin is further classified into two types based on the nature of the reducing ends: the G-chain, containing a terminal d-glucose unit, and the M-chain, containing *O*-substituted d-mannitol at the termini. The proportion of M *versus* G chains varies among species, and in some cases, the M-chain is completely absent.^35^

The next class of macroalgae is red algae, which contains a variety of glycans. This section focuses on three primary types: carrageenan, agar, and mannan.

#### 2.1.4 Carrageenan

Carrageenan is a class of sulfated polysaccharides primarily composed of d-galactose units, found predominantly in red algae. Carrageenan exists in three main types: κ-carrageenan, ι-carrageenan, and λ-carrageenan. All three types share a general backbone of alternating α-(1 → 4)-d-galactose or 3,6-anhydro-d-galactose and β-(1 → 3)-d-galactose, with varying positions containing sulfate esters and 3,6-anhydro bridges.^36^ κ-Carrageenan consists of a disaccharide repeating unit of β-(1 → 3)-d-galactose-4-*O*-sulfate linked to α-(1 → 4)-3,6-anhydro-d-galactose. ι-Carrageenan contains a disaccharide repeating unit of β-(1 → 3)-d-galactose-4-*O*-sulfate linked to α-(1 → 4)-3,6-anhydro-d-galactose-2-*O*-sulfate. λ-Carrageenan is composed of a disaccharide repeating unit of β-(1 → 3)-d-galactose-2-*O*-sulfate linked to α-(1 → 4)-d-galactose-2,6-*O*-sulfate.^37^ The highest global production of carrageenan comes from species of *Eucheuma* and *Kappaphycus*, with *Kappaphycus alvarezii* being the primary producer of κ-carrageenan.^38^

#### 2.1.5 Agar

Agar is composed of two main components, agarose and agaropectin, which are found in the cell walls of red algae, particularly in species of *Gracilaria* and *Gelidium.*^39,40^ Agarose consists of a disaccharide unit called agarobiose, composed of α-(1 → 4)-3,6-anhydro-l-galactose and β-(1 → 3)-d-galactose. Agaropectin makes up a smaller portion of agar, shares a similar backbone but also contains additional modifications, such as methoxyl, sulfate, and pyruvate groups at various positions along the chain.^41^ The amount of agaropectin varies across species; for example, *Gracilaria* species generally contain more agaropectin than *Gelidium* species.^42^ Unlike carrageenan, agar contains 3,6-anhydro-l-galactose, whereas carrageenan contains 3,6-anhydro-d-galactose.

#### 2.1.6 Mannan

Mannans are important polysaccharides found in red seaweeds, exhibiting considerable diversity, including both α- and β-mannan forms. One prominent variant found in *Nemalion vermiculare* is α-(1 → 3)-d-mannan, often sulfated at the O-4 and O-6 positions, with a d-xylose residue branching from C-2.^43^ Another form, β-(1 → 4)-d-mannan, is found in the cuticle of *Porphyra umbilicalis.*^44^ These mannans, particularly the sulfated varieties, are crucial for the structural integrity and functional properties of the red seaweed cell wall, helping the organism withstand environmental stresses.

The final class of macroalgae is green algae that are primarily recognized for producing ulvan.

#### 2.1.7 Ulvan

Ulvan is a highly sulfated polysaccharide found in the extracellular matrices of green algae, particularly within *Ulva* species. Ulvan consists of two main types of repeating disaccharide units, type A and type B, commonly referred to as ulvanobiuronic acids. Type A is characterized by a repeating unit of β-(1 → 4)-d-glucuronic acid linked to α-(1 → 4)-l-rhamnose-3-*O*-sulfate (A3S), while type B consists of α-(1 → 4)-l-iduronic acid linked to α-(1 → 4)-l-rhamnose-3-*O*-sulfate (B3S). In some variants, the uronic acids are replaced by d-xylose, forming ulvanobioses, which include β-(1 → 4)-d-xylose linked to α-(1 → 4)-l-rhamnose-3-*O*-sulfate (U3S) or β-(1 → 4)-d-xylose-2,3-*O*-sulfate linked to α-(1 → 4)-l-rhamnose-3-*O*-sulfate (U2′S,3S).^45^ The occurrence of these ulvan variants is highly species-dependent. For instance, ulvanobiuronic acids are more widely distributed across *Ulva* species, whereas ulvanobioses are primarily found in *Ulva rigida* from the Canary Islands and France.^45^

### 2.2. Glycans in marine invertebrates and vertebrates

Marine invertebrates and vertebrates produce glycans of significant interest, such as chitin, chitosan, and glycosaminoglycans that have been studied extensively. Unlike glycans from macroalgae and other organisms discussed in this review, the structures of these glycans are generally well-preserved across species, making them valuable for scientific research and applications.

#### 2.2.1 Chitin

Chitin is a linear polysaccharide composed of β-(1 → 4)-*N*-acetyl-d-glucosamine residues. It is commonly found in the exoskeletons of marine invertebrates, such as crustaceans (shellfish, crabs, shrimps),^46^ as well as in the cell walls of terrestrial fungi and exoskeletons of insects.^47^ The structure of chitin remains largely consistent across species, from terrestrial organisms to marine invertebrates, making it the second most abundant polysaccharide after cellulose.^48^

#### 2.2.2 Chitosan

Chitosan is formed through the deacetylation of chitin, resulting in repeating units of β-(1 → 4)-d-glucosamine and β-(1 → 4)-*N*-acetyl-d-glucosamine, with more than 50% deacetylation.^49^ Chitosan is obtained by first extracting chitin and then subjecting it to deacetylation.^50^

#### 2.2.3 Glycosaminoglycans (GAGs)

Glycosaminoglycans are linear, unbranched polysaccharides composed of disaccharide repeating units that carry a high negative charge. These polysaccharides are found in the extracellular matrices of mammalian cells, as well as those of marine invertebrates and vertebrates.^51^ GAGs are classified into four main groups based on their disaccharide repeating units: heparin/heparan sulfate (HS), chondroitin sulfate (CS)/dermatan sulfate (DS), keratan sulfate (KS), and hyaluronic acid (HA).

Heparin and heparan sulfate are composed of disaccharide units of (1 → 4)-α-*N*-acetyl-d-glucosamine linked to α-(1 → 4)-l-iduronic acid (heparin) or β-(1 → 4)-d-glucuronic acid (heparan sulfate).^52^ Heparin contains a high degree of sulfation, with modifications varying depending on the source. Sulfation commonly occurs on the amino group of the amino sugar and on O-6 of the glucosamine residue, while O-2 sulfonate groups are found on the uronic acids.^53^ Chondroitin sulfate consists of β-(1 → 4)-d-glucuronic acid linked to β-(1 → 3)-*N*-acetyl-d-galactosamine, with frequent sulfation at O-6 of the galactosamine residue. Dermatan sulfate, similar to chondroitin sulfate in backbone structure, differs by having l-iduronic acid instead of d-glucuronic acid residues.^54^ Keratan sulfate is composed of disaccharide repeating units of β-(1 → 3)-d-galactose linked to β-(1 → 4)-*N*-acetyl-d-glucosamine, with the sulfate group often present on O-6 of the glucosamine residue.^55^ Finally, hyaluronic acid is the only non-sulfated GAG. HA consists of a disaccharide unit of β-(1 → 4)-d-glucuronic acid linked to β-(1 → 3)-*N*-acetyl-d-glucosamine, forming an alternating structure with respect to the (1 → 4) and (1 → 3) linkages.^52^

### 2.3. Glycans in marine microorganisms

Marine microorganisms, namely fungi, bacteria, and microalgae, are key players in the oceanic carbon cycle. Marine fungi and bacteria, residing in the deep ocean, break down glycans into their chemical constituents.^6,56^ In contrast, microalgae, including diatoms and cyanobacteria, inhabit the ocean's surface where they fix carbon from the atmosphere and serve as a primary food source for marine organisms.^3^ These marine microbes contribute to the circulation of marine carbon by biosynthesizing and releasing glycans into the environment. This section provides an overview of the common types of glycans originating from these marine microorganisms.

#### 2.3.1 Glucan

Glucans are present in marine fungi and diatoms in the forms of α- and β-glucans. α-Glucans, commonly found in marine fungi, typically feature a backbone of α-(1 → 4)-d-glucose, with some fractions containing α-(1 → 6)-d-glucuronic acid at the non-reducing end.^57^ Meanwhile, β-glucans are predominantly composed of β-(1 → 3)-d-glucan residues, with lower amounts of β-(1 → 6)-d-glucan branching units. These β-glucans are frequently observed in marine diatoms.^58,59^

#### 2.3.2 Exopolysaccharides (EPS)

Exopolysaccharides are glycans secreted by marine microbes into their environment, primarily serving as a defence mechanism against extracellular stress.^60^ These glycans are composed of diverse monosaccharides, including common sugars like d-mannose, d-galactose, and d-glucuronic acid, and rare sugars such as 3-deoxy-d-*manno*-oct-2-ulosonic acid (Kdo) and deoxy sugars. EPS exhibit significant structural diversity, with modifications like acetylation, carboxymethylation, phosphorylation, and sulfation. They also feature a variety of glycosidic linkages, which can include branched, terminal, or linear motifs.^61^ EPS have been studied in a wide range of microbial species, including fungi, bacteria, and diatoms.^62^ For example, an EPS from the deep-sea bacterium *Vibrio alginolyticus* contains a tetrasaccharide unit with α-(1 → 3)-d-galacturonic acid-α-(1 → 4)-d-galacturonic acid-α-(1 → 3)-d-galacturonic acid-β-(1 → 3)-*N*-acetyl-d-glucosamine.^63^ In contrast, an EPS from the seawater bacterium *Pseudoalteromonas flavipulchra* features a trisaccharide unit composed of 4-*O*-acetylated-6-deoxy-l-talose, β-(1 → 3)-d-galactose, and α-(1 → 7)-Kdo.^64^

## 3. Industrial applications of marine glycans

Marine glycans play a significant role across numerous industrial sectors. This section will explore five primary applications of marine glycans: (1) biomedicine and pharmaceutical, (2) biotechnology and bioengineering, (3) cosmetics and skincare, (4) food and nutrition, and (5) agriculture and aquaculture. Each application area will highlight select examples from the three main groups of marine organisms. A more comprehensive listing is provided in Table 1. As research progresses, the applications of marine glycans continue to expand, unveiling new opportunities across various fields.

### 3.1. Biomedicine and pharmaceuticals

The biomedical and pharmaceutical industries continuously seek to improve existing therapeutics and explore novel treatments, driving the investigation of marine-derived glycans for their diverse therapeutic properties and potential to advance medicine.

#### 3.1.1 Marine glycans from macroalgae

Wang *et al.*^8^ demonstrated that fucoidan from brown algae alleviated chemotherapy-induced alopecia and enhanced chemotherapy efficacy. Jang *et al.*^65^ reported that λ-carrageenan displayed antiviral activity against influenza viruses and SARS-CoV-2. Meanwhile, Recalde *et al.*^66^ showed that over-sulfated mannans from the red alga *Nemalion helminthoides* had potent virucidal activity against herpetic and dengue viruses. Son *et al.*^67^ found that ulvan from *Ulva pertusa* reduced weight loss, activated immune cells, and increased cytokine secretion in immunosuppressed mice.

#### 3.1.2 Marine glycans from invertebrates and vertebrates

Solairaj *et al.*^68^ reported the anticancer potential of chitin-copper/silver nanocomposites against human breast cancer cells. Loutfy *et al.*^69^ demonstrated that chitosan nanoparticles incorporating silymarin were effective antiviral agents against SARS-CoV-2. In addition, Egea *et al.*^70^ highlighted the antioxidant and neuroprotective activities of chondroitin sulfate (CS) in neuroblastoma cells, suggesting CS's potential for treating neurodegenerative diseases.

#### 3.1.3 Marine glycans from microorganisms

Rizzi *et al.*^58^ isolated a β-glucan from the marine diatom *Conticribra weissflogii*, which enhanced macrophage activity without cytotoxicity against glioblastoma cells. Ghareeb *et al.*^71^ studied EPS from the marine bacterium *Streptomyces vinaceusdrappus*, which exhibited antioxidant, anti-inflammatory, anti-diabetic, anti-Alzheimer, antibacterial, and antibiofilm properties.

### 3.2. Biotechnology and bioengineering

In biotechnology and bioengineering, growing interest in new bio-based technologies motivates the exploration of marine-derived glycans, whose unique properties enable innovations in bioprinting, tissue engineering, and biosynthetic materials.

#### 3.2.1 Marine glycans from macroalgae

Norouzi *et al.*^11^ identified that a 4% w/v alginate concentration in silk fibroin bioink provided optimal accuracy for 3D printing scaffolds in bone tissue engineering. Bitencourt *et al.*^72^ developed carrageenan-based gels for dysphagia patients, noting that κ-carrageenan concentration impacts gel texture and printing performance. Pari *et al.*^73^ further reviewed the diverse applications of ulvan-based biomaterials in biotechnology.

#### 3.2.2 Marine glycans from invertebrates and vertebrates

Zheng *et al.*^74^ explored chitin's potential in 3D printing hydrogels for wound dressings, finding that β-chitin nanofiber concentration significantly influenced the quality of the printed scaffold, with 5–10 wt% yielding optimal performance. Ahmed *et al.*^75^ developed chitosan–MgO nanocomposites with antibacterial properties for leather, improving durability and resistance to environmental degradation. Lou *et al.*^76^ fabricated hyaluronic acid-collagen-based hydrogels to mimic the extracellular matrix in 3D cell cultures, enhancing cell spreading and fiber remodelling by adjusting HA concentration and crosslinking properties.

#### 3.2.3 Marine glycans from microorganisms

Teixeira *et al.*^77^ isolated an EPS from *Klebsiella oxytoca*, which effectively stabilized hydrophilic emulsions, displayed iron-chelating properties, improved viscosity, and was non-toxic to non-tumor cells. Gutiérrez *et al.*^78^ investigated an EPS from *Antarctobacter* sp. TG 22, which formed highly stable emulsions, outperforming non-marine EPS like xanthan gum and gum Arabic in stabilizing ability.

### 3.3. Cosmetics and skincare

The cosmetics industry leverages marine glycans for their bioactive properties as active ingredients in cosmetic products. These glycans are valued for their therapeutic potential and sustainable sourcing.^79^

#### 3.3.1 Marine glycans from macroalgae

Cheong *et al.*^80^ enhanced laminarin's bioactivity by introducing ester modifications, resulting in laminarin butyl esters with anti-glycation properties that prevent skin aging and promote skin whitening. Zhu *et al.*^81^ enzymatically degraded κ-carrageenan into tetrasaccharides, which maintained hydration in keratinocytes and reduced oxidative stress and inflammation. Don *et al.*^82^ developed a chitosan-ulvan film with enhanced tensile strength and bioactivities, including antioxidant effects, skin whitening, and selective toxicity to melanoma cells.

#### 3.3.2 Marine glycans from invertebrates and vertebrates

A chitosan-based face mask with *Achyranthes aspera* leaf extracts exhibited antibacterial, antioxidant, and anti-aging properties, while being non-toxic to mouse embryonic fibroblasts.^83^ Galvez-Martin *et al.*^84^ investigated a hyaluronic acid matrix with dermatan sulfate, chondroitin sulfate, and collagen, demonstrating regenerative effects on fibroblasts and keratinocytes, alongside moisturizing, antioxidant, and anti-aging benefits for both oral and topical applications.

#### 3.3.3 Marine glycans from microorganisms

Tseng *et al.*^85^ found that polysaccharide extracts from the cyanobacterium *Nostoc commune* possessed anti-allergic and skin-protective properties, improving skin elasticity and flexibility. Additionally, a wide range of marine EPS have been featured in several cosmeceutical patents,^86^ showcasing anti-aging, anti-inflammatory, and wrinkle reducing activities.

### 3.4. Food and nutrition

Marine glycans are significant contributors to the food industry, utilized to elevate nutrient content and food preservation qualities.

#### 3.4.1 Marine glycans from macroalgae

Moroney *et al.*^15^ observed that laminarin and fucoidan extracted from *Laminaria digitata* reduced iron-induced lipid oxidation in pork liver tissues, suggesting their antioxidant potential in high protein foods. Menaka and Wijesekara^87^ explored agar from *Gracilariopsis longissimi* as a plant-based gelatin alternative for food jellies, highlighting its viability as a gelling agent. Morelli *et al.*^88^ validated ulvan as an emulsifying agent in functional food formulations, particularly in sustainable oil and water emulsions for soft drinks.

#### 3.4.2 Marine glycans from invertebrates and vertebrates

Yin *et al.*^213^ investigated chitin's role in stabilizing Pickering emulsions, using chitin nanowhiskers to encapsulate flavor compounds in essential oils and reduce lipid oxidation. Paulose and Chakraborty^254^ extracted a sulfated glycosaminoglycan-like heteropolysaccharide from the octopus *Cistopus indicus*, which enhanced glucose uptake in adipocytes, suggesting its potential as a bioactive ingredient in functional foods for managing type-2 diabetes.

#### 3.4.3 Marine glycans from microorganisms

Gan *et al.*^267^ identified a novel EPS from *Halomonas saliphila* strain LCG169T, which exhibited oil-capturing, foaming, and emulsifying properties, making it a potential bioemulsifier for oils like olive or sunflower oil. Sran *et al.*^268^ discovered an EPS from *Rhodobacter johrii* that formed a thermally stable bioemulsifier, ideal for improving texture and stability in food products.

### 3.5. Agriculture and aquaculture

Marine glycans are recognized for their efficacy in boosting productivity and promoting healthy ecosystems.

#### 3.5.1 Marine glycans from macroalgae

Aboulella *et al.*^111^ examined alginate's role in water management, synthesizing hydrogels that desalinated water and increased potassium concentrations for nutrient-rich irrigation. Thye *et al.*^171^ showed λ-Carrageenan improves nutrient uptake and cell homeostasis in banana plants to enhance growth. Velho *et al.*^202^ observed that ulvan can boost plant resistance to pathogenic fungi by upregulating genes that strengthen cell walls.

#### 3.5.2 Marine glycans from invertebrates and vertebrates

Liu *et al.*^235^ demonstrated chitosan, combined with enzyme-induced carbonate precipitation, protected red mud from wind erosion by enhancing carbonate production and formed a durable crust layer. Njimou *et al.*^219^ synthesized chitin–MnO_2_–alginate nanoparticles for wastewater treatment and achieved effective adsorption of Cd(ii) and Pd(ii) in a spontaneous and endothermic process.

#### 3.5.3 Marine glycans from microorganisms

Reyes-Becceril *et al.*^260^ isolated a β-(1 → 3)-glucan with (1 → 6) branching from the marine yeast *Debaryomyces hansenii*; the glycan enhanced fish health by exhibiting antioxidant activities and promoting intestinal health without causing histopathological damage. Similarly, Perveen *et al.*^259^ studied β-(1 → 3)-glucan from the microalga *Euglena gracilis* and reported that this glucan increased enzyme responses, upregulated innate immune genes, and exhibited dose-dependent antiparasitic activity in marine crabs against *Mesanophrys* spp.

## 4. Extraction of marine glycans

### 4.1. Enzymatic degradation of marine glycans

The enzymatic degradation of marine glycans is essential to global carbon cycling,^23^ organic matter turnover, and biogeochemical processes, reinforcing ocean productivity and ecological balance.^272^ Heterotrophic microorganisms biosynthesize a diverse array of specialized carbohydrate-active enzymes (CAZymes) that catalyze the hydrolysis of specific glycosidic bonds in polysaccharides. These enzymes include, but are not limited to, glycoside hydrolases (GHs), agaroses, sulfatases, carrageenases, alginate lyases, chitinases, amylases, lipases, phytases and proteases.^273^ They are systematically categorized into families based on their structures and functions, as detailed in the CAZymes database (https://www.cazy.org). This diverse array of CAZymes efficiently liberates valuable monosaccharides and bioactive molecules from marine biomass,^274,275^ including agarose, alginate and sulfated polysaccharides derived from seaweeds; chitin and chitosan from crustaceans; and collagen and glycosaminoglycans from fish. A deeper understanding of the enzymatic mechanisms unlocks significant potential for biotechnological and industrial applications (Table 1).^46,276^

### 4.2. Enzymatic extraction of marine glycans

Different enzymatic methods have been explored to enhance the efficiency of marine polysaccharide degradation. Conventional extraction often employs harsh conditions that can diminish the target molecule's functional attributes. In contrast, enzymatic extraction with specific hydrolyses has emerged as a promising approach for augmenting product yield, preserving bioactive properties, and minimizing environmental impact.

A comprehensive analysis of the extraction, modification, degradation, and bioactivity of pivotal marine polysaccharides, encompassing agar, fucoidan,^28^ ulvan,^274,277^ carrageenan,^161^ alginate, chitin^278,279^ and chitosan focused on elucidating the underlying enzymatic mechanisms.^24,280–282^ The biodiversity of CAZymes involved in marine polysaccharide degradation and their ecological roles have been studied.^24,283^ Liu *et al.*^284^ highlighted recent advancements in enzymatic, chemical, and physical methodologies for the depolymerization of fucoidan into low-molecular weight fucoidan and fuco-oligosaccharides. Wu *et al.*^285^ identified a broad-specificity, high-thermostability chitinase (AfChi28) from the marine fungus *Aspergillus fumigatus* df347, positioning AfChi28 as a potential biocatalyst for chitin oligosaccharide production. Additionally, highly specific glycoside hydrolases derived from marine flavobacteria were characterized and demonstrated their efficacy in analysing laminarin from diatoms and seawater samples.^20^ Challenges associated with the standardization of fucoidan preparations and the potential applications of fucoidanases in pharmaceutical and nutraceutical sectors have been explored.^286^ Analytical methodologies are key to the elucidation of structural features and molecular composition of compounds post-extraction, particularly in the characterization of complex marine polysaccharides.^19,22^

### 4.3. Challenges in marine glycans biodegradation by enzymes

**Fig. 4:**
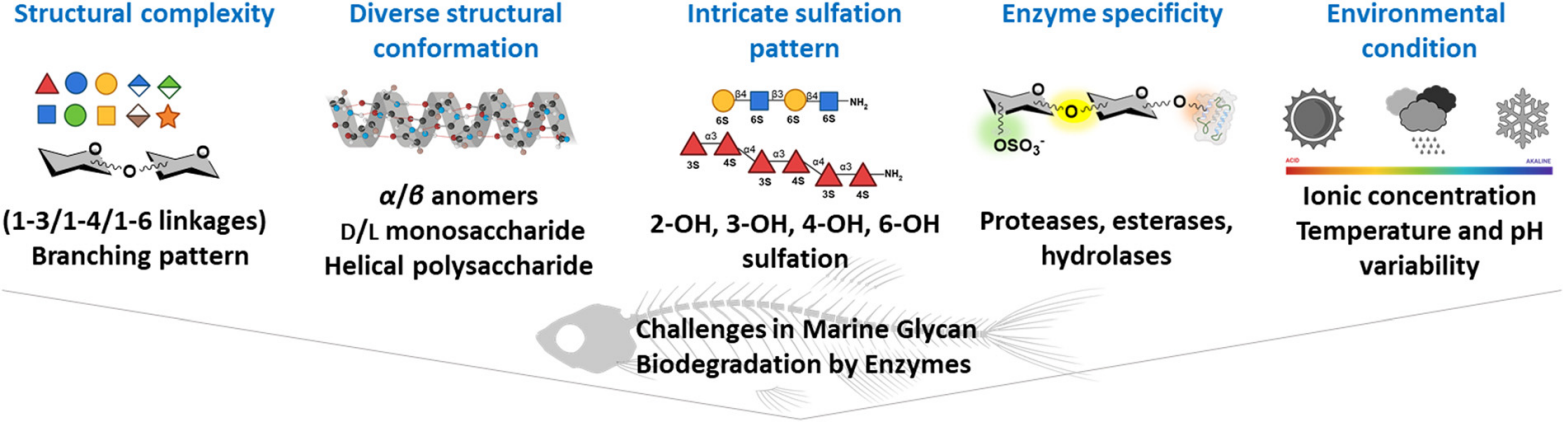
Challenges in marine glycan biodegradation by enzymes.

The intricate structural modifications of marine polysaccharides confer significant resistance to enzymatic degradation, presenting a major challenge for developing a comprehensive marine glycomic workflow.^24,287^ Structural complexity, unique monosaccharide compositions, distinct glycosidic linkages, heterogeneous molecular weights, diverse conformational architectures, intricate sulfation patterns, and limited enzymatic efficiency are challenging. The high variability of marine polysaccharides across species further complicates the establishment of standardized degradation and characterization methods. Understanding these complexities is essential for effectively harnessing the potential of marine polysaccharides (Fig. 4).

#### 4.3.1 Structural complexity of marine glycans

Marine glycans exhibit remarkable structural complexity and monosaccharide variability, demanding a wide range of CAZymes for enzymatic degradation.^272^

A single CAZyme targets specific linkages, but complex polysaccharides necessitate a corresponding set of enzymes, each specialized for a particular linkage. Fucoidan, a highly heterogeneous sulfated polysaccharide derived from brown algae, features complex branching, diverse linkages, and variable sulfation patterns. As a result, fucoidan degradation requires 284 putative fucoidanases, glycoside hydrolases (GHs), sulfatases, and carbohydrate esterases, as shown in *Verrucomicrobium* bacteria.^288^ In *Lentimonas* sp. CC4, 100 enzymes are utilized to break down fucoidan,^289^ emphasizing the extensive enzymatic machinery needed to address its structural heterogeneity. In contrast, less complex β-glucans like laminarin are degraded rapidly by just two to three enzymes.^290^

The breakdown of carrageenan is a complex process, requiring the coordinated action of multiple enzymes to overcome the structural intricacies of sulfated galactan from red algae.^291^ Side chains limit the activity of endo-acting enzymes due to steric hindrance, hindering the complete hydrolysis of polysaccharides like laminarin.^21,292^ Specifically, GH16 and GH17 enzymes exhibit different specificities towards laminarin, with GH17 enzymes showing narrow specificity for non-decorated β-(1 → 3)-glucan stretches, suggesting that the presence of β-(1 → 6) side chains impede their activity.^20^ To fully degrade polysaccharides, bacteria require a unique enzyme for each distinct chemical bond between the building blocks, underscoring the need for a robust enzymatic repertoire to address the diversity of glycosidic linkages.^23^

#### 4.3.2 Intricate sulfation patterns

Many marine polysaccharides are sulfated, and their sulfation levels and patterns can significantly influence their biological activities and interactions with cell receptors (Table 1). Desulfation of marine sulfated galactofucans resulted in lower anti-thrombin binding compared to their sulfated counterparts.^293^ On other hand sulfate groups attached at various positions on monosaccharides (O-2, O-3, or O-4) increase negative charge and steric hindrance, hindering enzyme access. Consequently, the removal of sulfate groups by sulfatases is often necessary before glycoside hydrolases can effectively act on the glycan backbone.^23^ Sulfate groups are essential for the activity of the fucoidanase FFA, likely due to specific binding interactions with the enzyme, whereas for the fucoidanase from *Lambis* sp., sulfate groups interfered with enzyme hydrolysis.^286^ Thus, the presence or absence of sulfate groups, depending on the enzyme, can either promote or hinder activity, potentially by affecting the enzyme's ability to approach and bind the substrate.

#### 4.3.3 Diverse structural conformations

Glycosidic linkages buried within the three-dimensional network of marine polysaccharides due to folding, hydrogen bonding, or interaction with water and ions can influence their functionality, including interactions with complement enzymes.^272,294^ The polysaccharide conformation affects the accessibility of glycosidic bonds to hydrolyzing enzymes that are typically stereospecific and linkage-specific.^22^ Hence, variations in configurations require enzymes with corresponding specificities.^20^ Compact three-dimensional arrangements, resulting from extensive inter- and intramolecular interactions, can render polysaccharides highly resistant to enzymatic degradation.^294^ The endo-α-1,6-mannanase (ShGH76) from *Salegentibacter* sp. Hel_I_6, interacts with kinked oligomannan conformations, a structural feature specific to fungal α-1,6-mannans.^295^ This study highlights how the complexity and conformational flexibility of glycans challenge enzymatic breakdown efficiency.

#### 4.3.4 Enzyme specificity

The efficiency of CAZymes is constrained by their specificity for particular glycan structures.^23^ GH enzymes, including porphyranases and agarases that target sulfated galactans, possess highly substrate-specific active sites.^23^ The enzymatic degradation of microalgal cell walls also requires enzymes that are highly specific and versatile for effective bioconversion.^296^ Thus, finding the exact enzyme with the required specificity for a particular marine glycan is challenging, given the underexplored nature of marine environments and their microbial enzyme diversity.^272^

#### 4.3.5 Environmental conditions

As algal blooms mature, more complex polysaccharides become available, requiring enzymes with greater specificity and efficiency for their degradation.^297^ Seasonal shifts in CAZyme expression underscore this constraint: in spring, elevated β-(1 → 3)-glucosidase activity targets laminarin, while in winter, α-glucan-degrading enzymes dominate.^298^ Enzymatic degradation is also dependent on environmental factors such as pH. Fucoidanases isolated from marine invertebrates, including the molluscs *Haliotus* sp., *Mizuhopecten yessoensis*, and the sea urchin *Strongylocentrotus nudus*, showed peak activity in the pH range of 3.5–5. In contrast, fucoidanase from the marine bacterium *Formosa algae* KMM 3553T exhibited maximal activity over a wide pH range from 6.5 to 9.^286^ Another factor influencing enzymatic degradation is optimal temperature. For κ-carrageenase OUC-FaKC16A, the optimal temperature ranges from 30 to 100 °C; ι-carrageenases function best between 30 to 65 °C; and alginate lyases from *Pseudoalteromonas* species shows optimal activity at 25 °C to 55 °C.^282^ Ionic strength is also critical; κ-carrageenases require specific ionic conditions such as the presence of Na^+^ or Ca^2+^ for peak activity, and variations in these conditions significantly impact degradation rate and efficiency.^299^

### 4.4. Advances in enzyme engineering

Recent advancements in recombinant enzyme technologies have enabled the production of enzymes with enhanced specificity and catalytic efficiency, leading to improved precision in degradation, enhanced capability for detailed structural analysis, and increased yield of bioactive oligosaccharides from marine biomass.^276^ For instance, the use of recombinant cCgkA and cCglA enzymes for carrageenan hydrolysis demonstrated a 3.1-fold increase in efficiency compared to individual enzymes.^300^ These innovations support the extraction of bioactive compounds from marine polysaccharides, such as YCP, a mitogenic polysaccharide that enhances phagocytic activity,^301^ and fucoidan, which shows promise as a therapeutic agent for Alzheimer's disease.^302^

Combining enzyme-assisted extraction with ultrasound treatment has significantly boosted ulvan recovery from *Ulva fenestrata*, yielding up to 18% compared to enzymatic extraction alone.^274^ Ultrasound-assisted enzymatic extraction enhances polysaccharide yield, reduces extraction time, improves cell disruption, and preserves bioactivity under mild conditions.^303^

Side group modifications of carbohydrates increase the recalcitrance of algae to enzymatic degradation, prompting marine organisms, particularly bacteria, to evolve specific enzymes that can remove these modifications from the carbohydrate backbone before utilizing common CAZymes to hydrolyze the glycosidic bonds.^24^ Also, the significance of enzyme engineering, particularly alginate lyases, to improve alginate degradation and expand its potential use in sustainable agriculture has been reviewed.^304^

## 5. Automated glycan assembly

Automated glycan assembly (AGA) has revolutionized carbohydrate chemistry by enabling the rapid, efficient synthesis of complex oligosaccharides.^305,306^ This solid-phase synthesis technique allows for the programmable construction of oligo- and polysaccharides from orthogonally protected monosaccharide building blocks, ensuring regio- and stereoselective coupling. AGA optimizes the synthesis workflow by reducing purification steps and handling, providing a controlled environment for precise glycan assembly.^25^ This technology has broad applications across scientific disciplines, deepening our understanding of glycobiology and advancing therapeutic development. AGA enables the rapid and reproducible synthesis of complex structures, driving innovation in glycoscience and aiding the design of drugs, vaccines, and diagnostic tools that utilize glycans.^307–309^

### 5.1 Scope of AGA in marine glycan synthesis

**Fig. 5:**
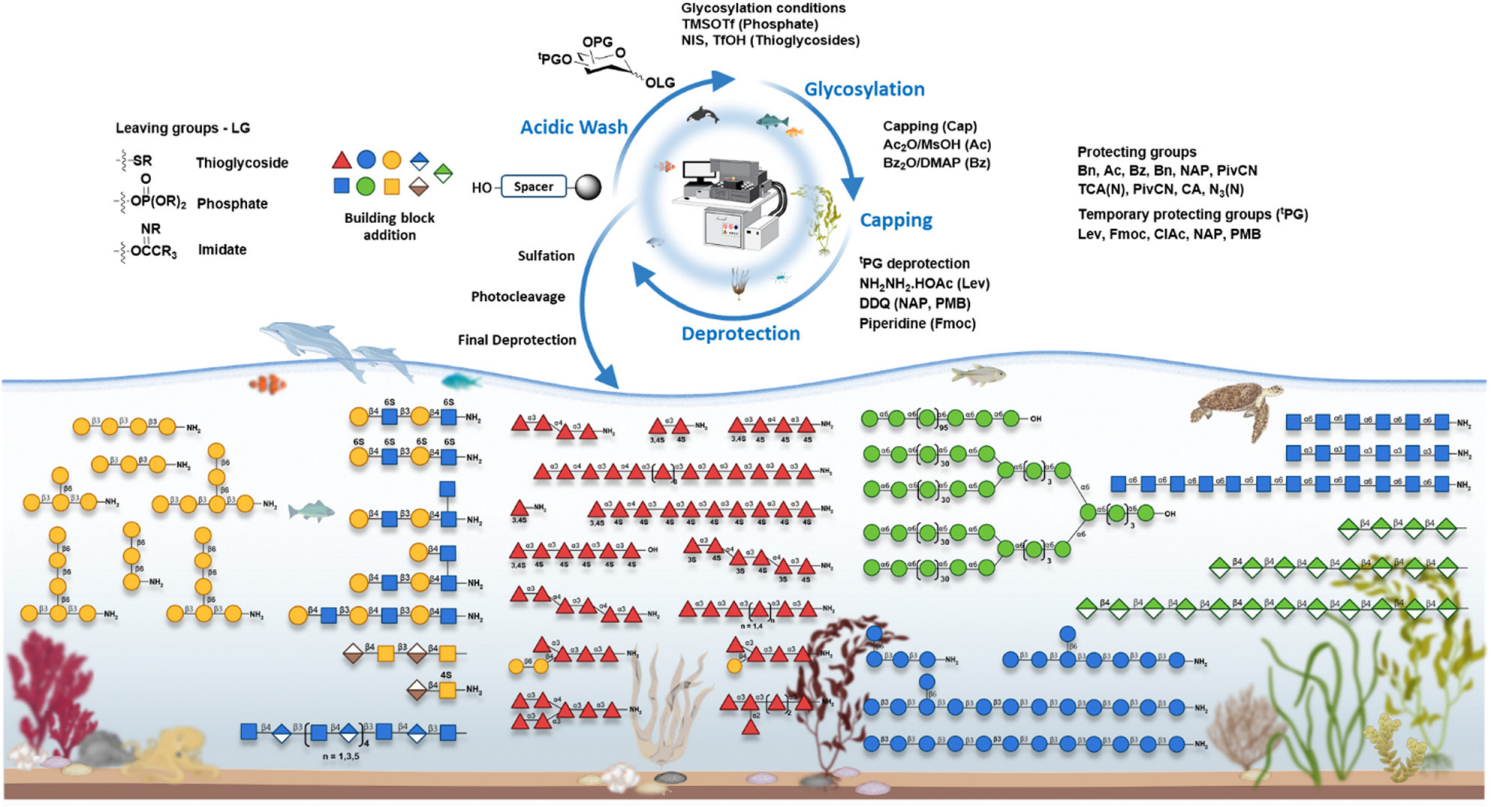
Scope of AGA in marine glycan synthesis.

Improvements in AGA methods and synthesis protocols have enabled the production of numerous glycans that represent the primary categories of marine carbohydrates with greater efficiency and accuracy. AGA provides a method to explore marine glycans, laying the groundwork for future innovations in marine-derived therapeutics and biomaterials (Fig. 5).

The rapid construction of linear and branched polysaccharides up to 100-mers using monosaccharides on an automated synthesizer provided the basis for constructing polysaccharides as large as 151-mers by a 31 + 30 + 30 + 30 + 30 block coupling.^26^ Production of complex sulfated polysaccharide primarily found in certain brown seaweeds and important for various biological processes, have been achieved through AGA, including the precise synthesis of galactofucan oligosaccharides.^305^

Research on the AGA of peptidoglycan backbone fragments provided methods that can be adapted for efficient, controlled synthesis of chitin and its derivatives, advancing the production of defined chitin oligosaccharides.^310,311^ AGA of oligo-β-glucans, key components of marine algae such as laminarin,^312^ and oligosaccharides related to arabinogalactan proteins contains β(1 → 3) and (1 → 6) linkages which can be further modified to synthesize carrageenan derivatives have been reported.^313^ GAGs represent a class of polysaccharides with diverse biological functions. AGA has emerged as a transformative technology for the chemical synthesis of GAGs, including keratan sulfate (KS),^314^ dermatan sulfates (DS),^315^ chondroitin sulfate (CS),^316^ and hyaluronic acid (HA),^317^ incorporating glucuronic acid, iudronic acid, and amino sugar building blocks. These studies demonstrate the capacity of AGA to precisely construct GAGs with controlled sulfation patterns, offering a robust platform for investigating the structure–function relationships of marine GAGs. Automated solid-phase synthesis has been successfully employed for the synthesis of β-mannuronic acid alginates, major components of the cell walls of algae, demonstrating the feasibility of constructing structurally defined marine glycans with challenging (1 → 2)-*cis*-mannosidic linkages.^318^

The stereo controlled formation of (1 → 2)-*cis*-glycosidic bonds is a general difficulty in oligosaccharide synthesis, particularly for fucoidan, which contains these challenging linkages. Additionally, the introduction of multiple sulfate groups at specific positions on the growing glycan chain adds considerable complexity. Careful design of protecting groups and compatible sulfation strategies that can be integrated into the automated process are required. The synthesis of algal fucoidan oligosaccharides, reaching lengths of up to 20-mers with diverse branching patterns and sulfate esters, reinforces AGA's capacity to handle the complexities of major marine polysaccharides.^305^

## 6. Future perspectives

AGA offers a powerful and versatile platform for synthesizing a diverse range of structurally defined marine glycans in a controlled laboratory setting. Marine glycans, such as heavily sulfated fucans and mannans, pose significant chemical challenges due to their complex structures. AGA platforms are continuously evolving to facilitate the rapid and reproducible synthesis of these glycans. Future advancements in AGA will focus on optimizing monosaccharide building blocks, glycan back-bone assembly, solid-phase chemistry, and sulfation methods to further expand the library of accessible glycans.

Synthetic glycans act as crucial tools for discovering new enzymes capable of degrading specific algal glycans like fucoidan and mannans. These enzyme cascades can then become tools for environmental detection and quantification of algal glycans, both in the laboratory and the ocean. By exposing microbes to this synthetic diversity, researchers can directly monitor microbe–glycan interactions, providing crucial insights into whether glycan diversity acts as a chemical barrier against degradation.

Synthetic glycans are essential for understanding their fundamental roles in ecological processes like carbon cycling, characterizing enzyme activities, developing new biocatalytic tools, and exploring their vast potential in industrial applications. Continued advancements in AGA methodologies will further enhance these capabilities, paving the way for ground-breaking discoveries in marine glycobiology.

## 7. Conclusions

Marine glycans are pivotal biomolecules in the oceanic carbon cycle, driving the sequestration of carbon in the deep ocean and contributing to the Earth's largest carbon sink. In addition to their ecological importance, marine glycans exhibit potent bioactivities leveraged by several industries, from medicine to agriculture. Nevertheless, the complexity of glycan structures hinders a fundamental understanding of their functions. Enzymatic extraction methods have made significant advancements in decoding these intricate glycans, yet they face inherent challenges due to incomplete knowledge of glycan structures and the enzymes involved in their biosynthesis and degradation. AGA has emerged as a promising solution, providing a platform for achieving structurally defined glycans that inform enzymatic studies on the molecular constituents responsible for their diverse bioactivities. Together, these complimentary approaches facilitate a synergistic investigation into this essential class of biomolecules, uncovering their ecological and biological roles in the coming decades.

## Conflicts of interest

The authors declare no conflicts of interest.

## Data Availability

No primary research results, software or code have been included and no new data were generated or analysed as part of this review.
